# Butorphanol Suppresses the Proliferation and Migration of Osteosarcoma by Promoting the Expression of piRNA hsa_piR_006613

**DOI:** 10.3389/fonc.2022.775132

**Published:** 2022-02-24

**Authors:** Pengfei Cui, Deqian Xin, Fu Li, Lin Deng, Yujie Gao

**Affiliations:** ^1^ Department of Anesthesiology, Yantaishan Hospital, Yantai, China; ^2^ Department of Anesthesiology, YanTai Yuhuangding Hospital, Yantai, China; ^3^ Department of Traumatology, Shu Guang Hospital Affiliated to Shanghai University of Traditional Chinese Medicine, Shanghai, China; ^4^ Department of Clinical Laboratory, YanTai Yuhuangding Hospital, Yantai, China

**Keywords:** butorphanol, osteosarcoma, piRNA, has_piR_006613, proliferation, migration

## Abstract

Butorphanol, a partial agonist of opioid receptor κ 1 receptor, can and is widely used as an analgesic drug to relieve moderate and severe pain in clinic. Osteosarcoma is one of the most common malignant bone tumor in adolescents under the age of 20. To our knowledge no study has investigated the effect of butorphanol on the proliferation of osteosarcoma cells. In this study, The proliferation of osteosarcoma cells was measured by CCK-8 and colony formation assays, and the migration of osteosarcoma cells were detected by scratch and transwell assays. The expression of piRNA was detected by RNA sequencing and real-time PCR. PiRNA mimics or inhibitors have been used to upregulate or inhibit piRNA expression in osteosarcoma cells, respectively. We found that butorphanol, at the concentration of 10ug/ml or higher, could significantly inhibit the proliferation and migration of osteosarcoma cells. Our resuslts indicated that butorphanol promoted the expression of piRNA hsa_piR_006613 and overexpression of piRNA hsa_piR_006613 inhibited the proliferation and migration of osteosarcoma cells. our study also showed that inhibition of the expression of piRNA hsa_piR_006613 could promote the proliferation and migration of osteosarcoma cells. Butorphanol played the regulatory role on osteosarcoma cells in dependent of piRNA hsa_piR_006613. Butorphanol was found to inhibit the proliferation and migration of osteosarcoma cells by promoting piRNA hsa_piR_006613 expression. Bioinformatics analysis showed that hsa_piR_006613 downregulated FN1 protein expression by binding with 3’-UTR of FN1 mRNA. In all, the present research indicated that butorphanol suppresses the proliferation of osteosarcoma by promoting the expression of piRNA hsa_piR_006613, which downregulated the expression of FN1. Has_piR_006613 may become a new therapeutic target for osteosarcoma.

## Introduction

Butorphanol is a synthetic mixed opioid receptor agonist and antagonist. Since it was listed in the United States in 1978, it has been listed in other countries worldwide and is widely used in clinical analgesia and adjuvant therapy (1). Butorphanol acts as an agonist for the κ receptor, exerts an excitatory effect on the δ receptor and shows different degrees of antagonism on the μ receptor (2). Clinically, it is mainly used in the adjuvant treatment of different types of pain, including moderate to severe pain during or after operation or acute headache and migraine (3). Its administration methods are very diverse, including intramuscular injection, intravenous injection, brachial plexus administration or epidural administration. However, few reports have investigated the effect of butorphanol on the proliferation and migration of tumors.

Osteosarcoma is a common malignant tumor in children worldwide (4). Osteosarcoma develops from osteoblasts and usually occurs during rapid bone growth. Primary tumors typically occur in long tubular bone, and a small part originates from axial bone. Typically, chemotherapy after surgical resection improves the efficacy of patients with local tumors, and the 5-year event-free survival (EFS) of treated patients is 65–70% (5). Unfortunately, most patients with osteosarcoma have metastasis at the time of diagnosis, and metastasis often occurs in patients with nonmetastatic osteosarcoma for the first time. Osteosarcoma metastasis usually occurs in the lung, followed by other bones. Chemotherapy, with or without surgical resection, has little effect on metastatic osteosarcoma, and the 5-year productivity of these patients is quite low. Butorphanol has been used for postoperative analgesia of various tumors, but the effect of butorphanol on osteosarcoma cells has not been reported.

Piwi interaction RNA, also known as piRNA, is differentially expressed in many solid tumors, including breast cancer, pancreatic cancer, liver cancer, lung cancer and gastric cancer (6, 7). Although few studies have investigated the functions of piRNAs in human cancer, piRNAs play a critical role in the development of cancer and are expected to be markers of tumor diagnosis and prognosis (8). At present, there are more than 3000 confirmed piRNAs in humans, and there are still a large number of undetected and unverified piRNAs (9). Till now, no study is available on the regulatory effect of piRNAs on osteosarcoma cells.

In this study, we investigated the regulatory effect of butorphanol on osteosarcoma and the expression of piRNA and explored the effect of piRNA on the function of osteosarcoma to provide new therapeutic methods and targets for osteosarcoma treatment.

## Methods

### Cell Culture and Treatment

Osteosarcoma cell lines MG63 and U2OS purchased from National Collection of Authenticated Cell Cultures and were cultured in high glucose DMEM supplemented with 10% fetal bovine serum and 1% penicillin/streptomycin antibiotics. The cells were cultured in an incubator under saturated humidity, with a CO_2_ concentration of 5% and a temperature of 37°C.

### CCK-8 Assay

The cells were inoculated into 96-well plates at a density of 1000/well, and 5 parallel samples were set in each group. The cells were cultured in a 5% CO2 incubator at 37°C, and cell proliferation was detected after 12, 24, 48 and 72 hours. Before detection, the cell culture medium was discarded, 100 µl of complete medium containing CCK-8 (#CK04, Dojindo) (including 10 µl of CCK-8) was added to each well, and the plate was placed in the incubator for 2 hours. The 96-well plate was placed in a multifunctional microplate reader to detect the absorbance at 450 nm.

### Scratch Assay

A horizontal straight line was drawn on the bottom of each well of a 6-well plate using a marker pen. Five parallel lines were drawn on the back of each hole. The transfected cells were inoculated into 6-well plates, and different cell quantities were collected according to the cell volume and growth rate. After 24 hours, the cell confluence was approximately 90%. Using a 1 ml pipette, a scratch was made perpendicular to the straight line at the bottom, followed by washing with PBS three times to remove the floating cells. Serum free DMEM medium was added, and the cells were incubated at 37°C in 5% CO2. Images were acquired at fixed points for 0 h and 24 h and compared.

### Colony Formation Assay

When the cell confluence in the culture dish was 80% ~ 90%, trypsin was added to digest the cells, complete culture medium was added to neutralize trypsin, and the supernatant was centrifuged and resuspended. Six-well plates were inoculated at a density of 400 cells/well, and then complete medium was added to 2 ml. The cells were then cultured in a 5% CO_2_ incubator at 37°C for 14 days. Next, the supernatant was discarded, the cells were washed with PBS, and then the cells were fixed with 2 ml of 4% paraformaldehyde for 30 min. After that, crystal violet dye solution was added, followed by incubation for 20 min. The six-well plate was then inverted to dry. A piece of white paper was placed at the bottom of the 6-well plate as the photo background, photos were taken, and colonies visible to the naked eye were counted.

### Transwell Assay

The chamber (#3428, Corning) was placed in the culture plate, 300 µl of serum-free medium was added to the upper chamber, and the plate was placed in the incubator for prewarming. The cells were digested, washed with PBS and resuspended twice to completely remove the serum. The serum-free medium was then resuspended and counted. In total, 20000 cells were inoculated into the upper chamber, the total liquid volume of the upper chamber was supplemented to 500 µl, and 1.5 ml of DMEM containing 2.5% FBS was injected into the lower chamber to remove the bubbles under the chamber. After culture, the plate was removed, the culture medium was discarded, the cells were fixed with 4% paraformaldehyde for 10 minutes and then stained with crystal violet for 10 minutes. After that, the upper ventricular cells were removed with a cotton swab, and the migrating cells were counted under the microscope.

### piRNA Sequence

MG63 cells were divided into two groups: control and butorphanol groups. The cells in the butorphanol group were treated with 10 μg/ml of butorphanol for 24 hours. The cells were collected with TRIzol reagent(#B610409,Sangon Biotech) for piRNA sequencing analysis, which was performed by SANGON Biotech (Shanghai) Co., Ltd.

### Real-Time PCR

MG63 cells were collected, total RNA was extracted, RNA concentration was measured, and DNA was removed. The RNA was then reverse transcribed into cDNA (the procedure was performed according to the instructions of the Takara Kit, #RR036A). The reaction conditions were as follows: 37°C for 15 min, 85°C for 5 s, and a 4°C infinite cycle. PCR amplification was performed using cDNA as a template and piRNA-specific primers. The PCR conditions were as follows: 95°C for 30 s, 95°C for 5 s, and 60°C for 34 s, for a total of 40 cycles. The relative expression was calculated using the 2^-ΔΔCT^ method. The primer used were listed in **Supplementary Table S1**.

### Ectopic Tumorigenesis in Nude Mice

The collected cells were in good condition. When the cells were 80% confluent, they were washed with PBS, digested with trypsin, neutralized with serum-containing medium, collected into a centrifuge tube, and centrifuged at 1100 rpm for 3 min. Next, complete medium was added, and the cell density was adjusted to 1×10^7^cell/ml. After disinfecting the back skin of nude mice, they were injected subcutaneously with 0.2 ml of the cell suspension. The nude mice were observed every day, and tumor growth was measured once a week.

### Western Blot

Protein lysate was acquired with RIPA and supernatant was collected with 30 minutes reaction on ice and 15 minutes centrifugation at 13000 rpm. The protein concentration was determined by BCA Protein Assay Kit (#NCI3225CH, Pierce, Illinois, USA). Twenty microgram of total protein was resolved by SDS-PAGE, followed by transferring onto a PVDF membrane (#ISEQ00010, Millipore Billerica, USA). The membranes were blocked in TBSTcontaining 5% non-fat milk and immunoblotted with FN1 (#ab268020, Abcam,1:1000) and GAPDH (#ab8245, Abcam,1:2000) and detected using HRP-labeled secondary antibody.

### Statistical Analysis

SPSS version 20.0 software (SPSS Inc., Chicago, IL) was used for data analysis. The results were described as means ± standard deviation. To compare two groups, t-test was applied; one-way ANOVA was used to compare the differences between multiple groups. P < 0.05 was considered statistically significant. “*” indicated a significant difference (p<0.05), and “**” indicated an extremely significant difference (p<0.01).

## Results

### Butorphanol Suppresses the Proliferation and Migration of Osteosarcoma

**Figure 1:**
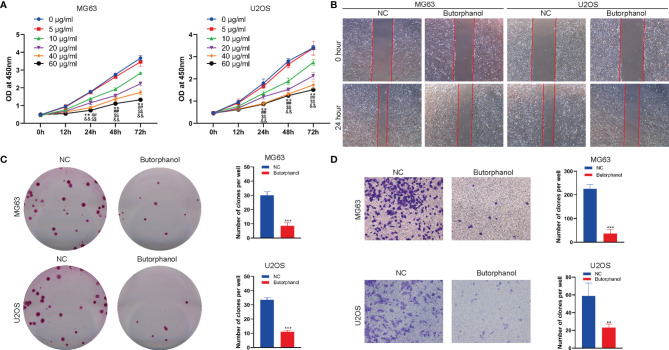
Butorphanol suppresses the proliferation and migration of osteosarcoma. **(A)** CCK-8 assay to detect the viability of MG63 and U2OS cells incubated with 0, 5, 10, 20, 40 and 60 μg/ml of butorphanol for 0, 12, 24, 48 h and 72 h ** indicates the 10 μg/ml group versus 0 μg/ml group (p<0.01), ^##^ indicates the 20 μg/ml group versus 0 μg/ml group (p<0.01), ^&&^ indicates the 40 μg/ml group versus 0 μg/ml group (p<0.01), and ^$$^ indicates the 60 μg/ml group versus 0 μg/ml group (p<0.01). **(B)** Scratch assay of the migration of MG63 and U2OS cells treated with 10 μg/ml of butorphanol for 24 h **(C)** Colony formation assay of the proliferation of MG63 and U2OS cells treated with 10 μg/ml of butorphanol. ** indicates p<0.01 and *** indicates p<0.001. **(D)** Transwell assay of the migration of MG63 and U2OS cells treated with 10 μg/ml of butorphanol for 24 h ** indicates p<0.01, and *** indicates p<0.001.

To investigate detect the effect of butorphanol on the function of osteosarcoma cells, we first detected the effect of butorphanol on the viability of MG63 and U2OS osteosarcoma cells using the CCK-8 assay. The CCK-8 assay results showed that butorphanol significantly inhibited the viability of MG63 and U2OS cells when the concentration of butorphanol was 10 μg/ml or higher(**Figure 1A**). Scratch tests found that pretreatment of MG63 and U2OS cells with 10 μg/ml of butorphanol significantly inhibited the migration of MG63 and U2OS cells (**Figure 1B**). The colony formation assay revealed that 10 μg/ml of butorphanol significantly inhibited the proliferation of MG63 and U2OS osteosarcoma cells (**Figure 1C**). The Transwell assay showed that pretreatment of MG63 and U2OS cells with 10 μg/ml of butorphanol significantly inhibited the migration of MG63 and U2OS cells (**Figure 1D**). Together, these results indicated that butorphanol inhibited the proliferation, migration of osteosarcoma cells.

### Butorphanol Promotes the Expression of piRNA hsa_piR_006613

**Figure 2:**
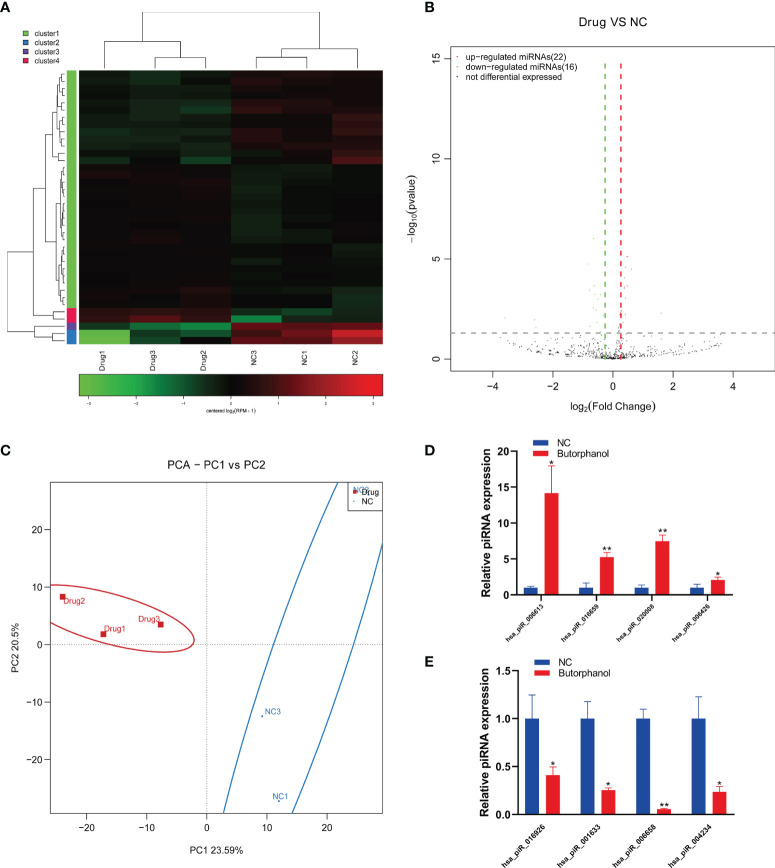
Butorphanol promotes the expression of piRNA hsa_piR_006613. **(A)** Heatmap of the expression of piRNA in MG63 cells treated with 10 μg/ml of butorphanol for 24 hours. **(B)** Volcano analysis of the expression of piRNA in MG63 cells treated with 10 μg/ml of butorphanol for 24 hours. **(C)** PCA of piRNA expression in MG63 cells treated with 10 μg/ml of butorphanol for 24 hours. **(D)** Real-time PCR of the upregulated expression of piRNA in MG63 cells treated with 10 μg/ml of butorphanol for 24 hours. **(E)** Real-time PCR showed the downregulated expression of piRNA in MG63 cells treated with 10 μg/ml of butorphanol for 24 hours. * indicates p<0.05, and ** indicates p<0.01.

To further study the mechanism of butorphanol in regulating osteosarcoma cells and the regulation of piRNA in osteosarcoma cells, we analyzed piRNA expression in the existence of butorphanol in osteosarcoma cells based on the piRNA sequence. We found that Butorphanol regulated the expression of different piRNAs in MG63 cells (**Figures 2A–C**). Real-time PCR showed that butorphanol promoted the expression of hsa_piR_006613, hsa_piR_016659, hsa_piR_020008 and hsa_piR_006426 and inhibited the expression of hsa_piR_016926, hsa_piR_001633, hsa_piR_006658 and hsa_piR_004234 in MG63 cells (**Figures 2D, E**).

### piRNA hsa_piR_006613 Overexpression Inhibits the Proliferation and Migration of Osteosarcoma

**Figure 3:**
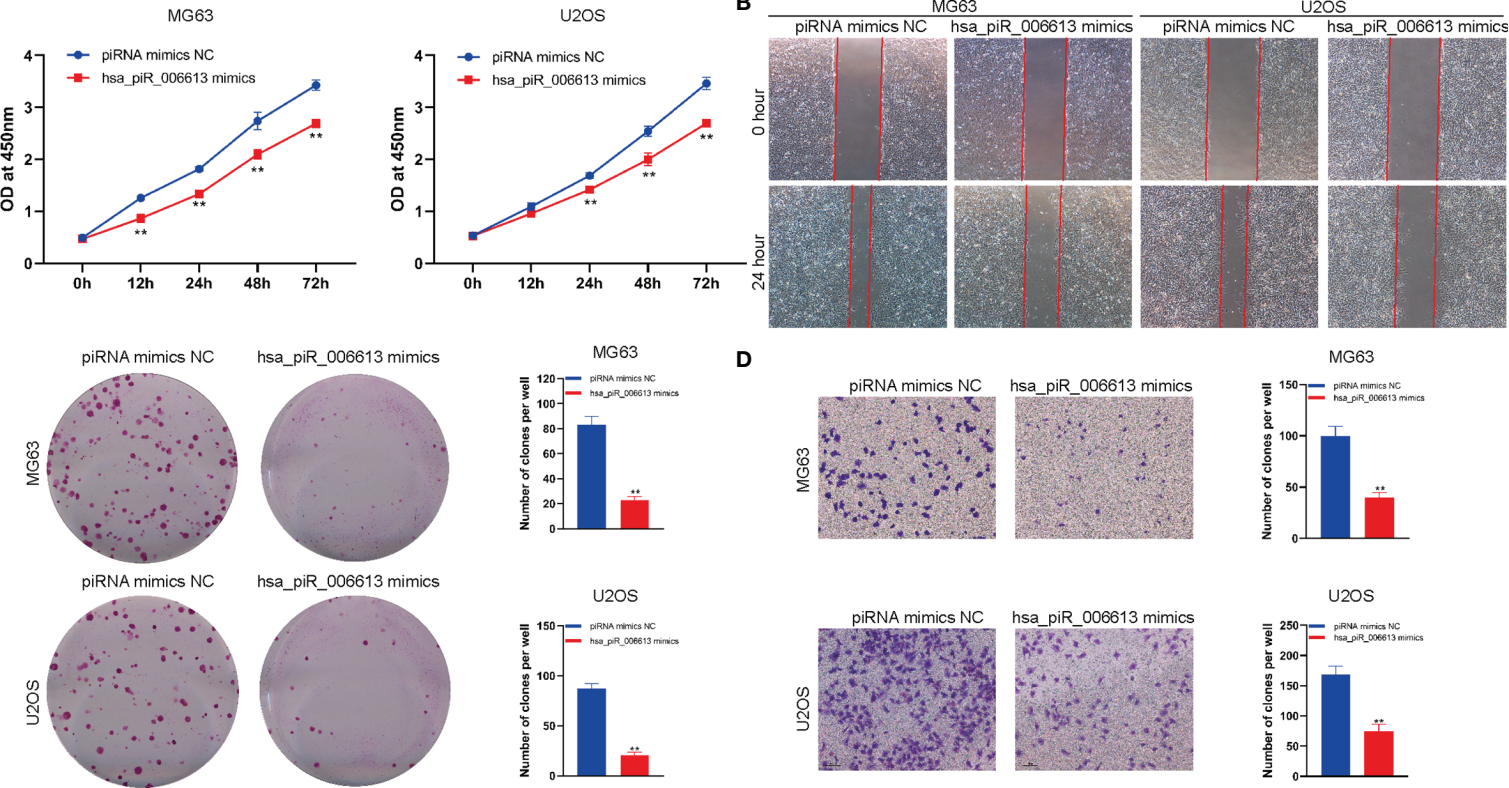
piRNA hsa_piR_006613 overexpression inhibits the proliferation and migration of osteosarcoma. **(A)** CCK-8 assay of the viability of MG63 and U2OS cells transfected with hsa_piR_006613 mimics or piRNA mimics NC. **(B)** Scratch assay of the migration of MG63 and U2OS cells transfected with hsa_piR_006613 mimics or piRNA mimics NC. **(C)** Colony formation assay of the proliferation of MG63 and U2OS cells transfected with hsa_piR_006613 mimics or piRNA mimics NC. **(D)** Transwell assay of the migration of MG63 and U2OS cells transfected with hsa_piR_006613 mimics or piRNA mimics NC. ** indicates p<0.01.

To detect the effect of hsa_piR_006613 on the function of osteosarcoma cells, we used hsa_piR_006613 mimics to upregulate the expression of hsa_piR_006613 in osteosarcoma cells. We first detected the effect of hsa_piR_006613 on the viability of MG63 and U2OS osteosarcoma cells using the CCK-8 assay. Overexpression of hsa_piR_006613 significantly inhibited the viability of MG63 and U2OS cells (**Figure 3A**). Scratch tests found that hsa_piR_006613 overexpression significantly inhibited the migration of MG63 and U2OS cells (**Figure 3B**). A colony formation assay found that hsa_piR_006613 overexpression significantly inhibited the proliferation of MG63 and U2OS osteosarcoma cells (**Figure 3C**). The Transwell assay showed that MG63 and U2OS cells with hsa_piR_006613 overexpression could significantly inhibit the migration of MG63 and U2OS cells (**Figure 3D**). has_piR_006613 overexpression inhibited the proliferation and migration of osteosarcoma cells.

### Inhibition of the Expression of piRNA hsa_piR_006613 Suppresses the Proliferation and Migration of Osteosarcoma

**Figure 4:**
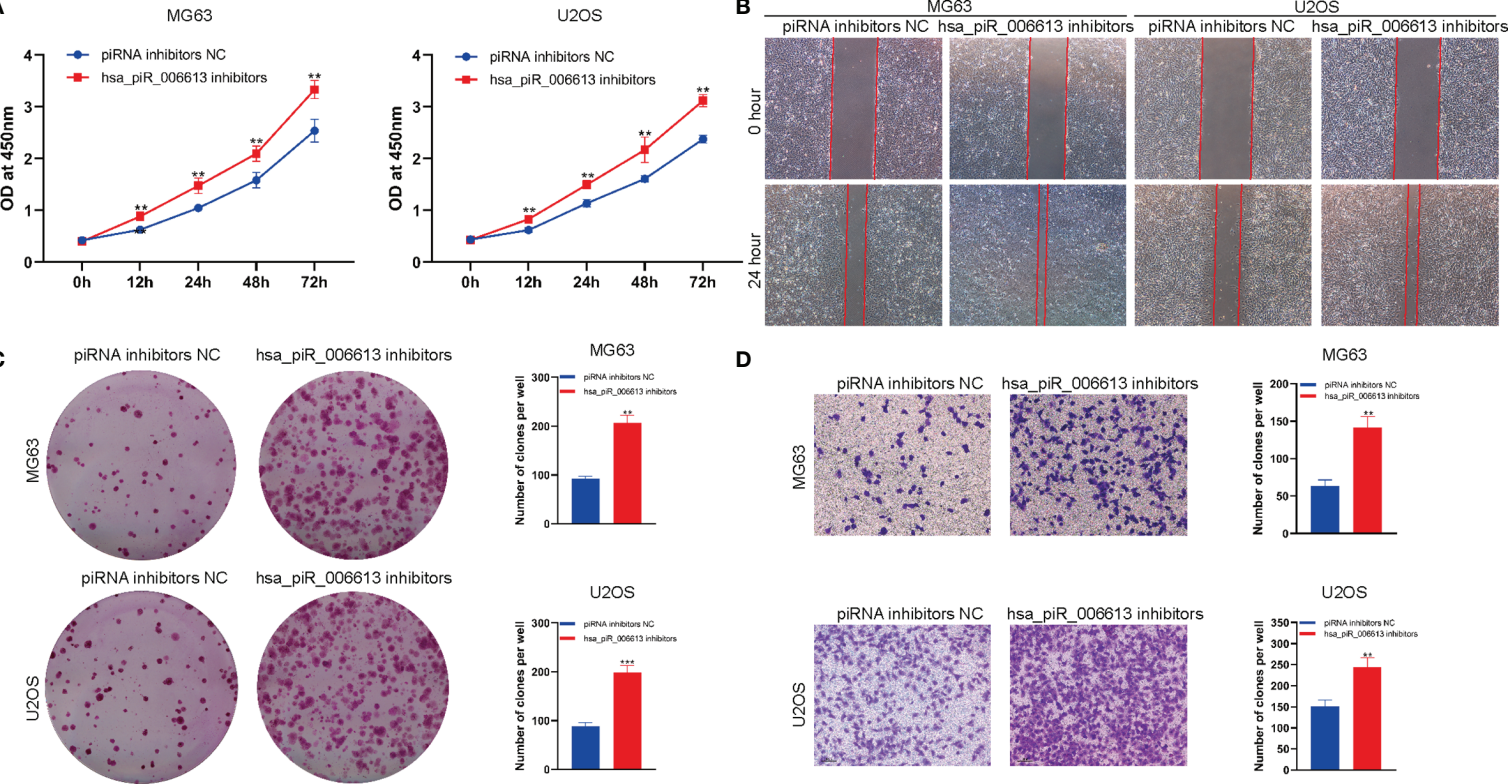
Inhibition of the expression of piRNA hsa_piR_006613 suppresses the proliferation and migration of osteosarcoma. **(A)** CCK-8 assay to detect the viability of MG63 and U2OS cells transfected with hsa_piR_006613 inhibitors or piRNA inhibitor NC. **(B)** Scratch assay of the migration of MG63 and U2OS cells transfected with hsa_piR_006613 inhibitors or piRNA inhibitor NC. **(C)** Colony formation assay of the proliferation of MG63 and U2OS cells transfected with hsa_piR_006613 inhibitors or piRNA inhibitor NC. **(D)** Transwell assay of the migration of MG63 and U2OS cells transfected with hsa_piR_006613 inhibitors or piRNA inhibitor NC. ** indicates p<0.01, and *** indicates p<0.001.

We used hsa_piR_006613 inhibitors to suppress the function of hsa_piR_006613 in osteosarcoma cells. The CCK-8 assay showed that hsa_piR_006613 inhibitors significantly promoted the viability of MG63 and U2OS cells (**Figure 4A**). Scratch tests found that hsa_piR_006613 inhibitors significantly promoted the migration of MG63 and U2OS cells (**Figure 4B**). The colony formation assay showed that hsa_piR_006613 inhibitors significantly increased the proliferation of MG63 and U2OS osteosarcoma cells (**Figure 4C**). The transwell assay showed that MG63 and U2OS cells treated with hsa_piR_006613 inhibitors significantly inhibited the migration of MG63 and U2OS cells (**Figure 4D**). hsa_piR_006613 inhibitors promoted the proliferation and migration of osteosarcoma cells.

### Butorphanol Regulates the Proliferation and Migration of Osteosarcoma in a piRNA hsa_piR_006613-Dependent Manner

**Figure 5:**
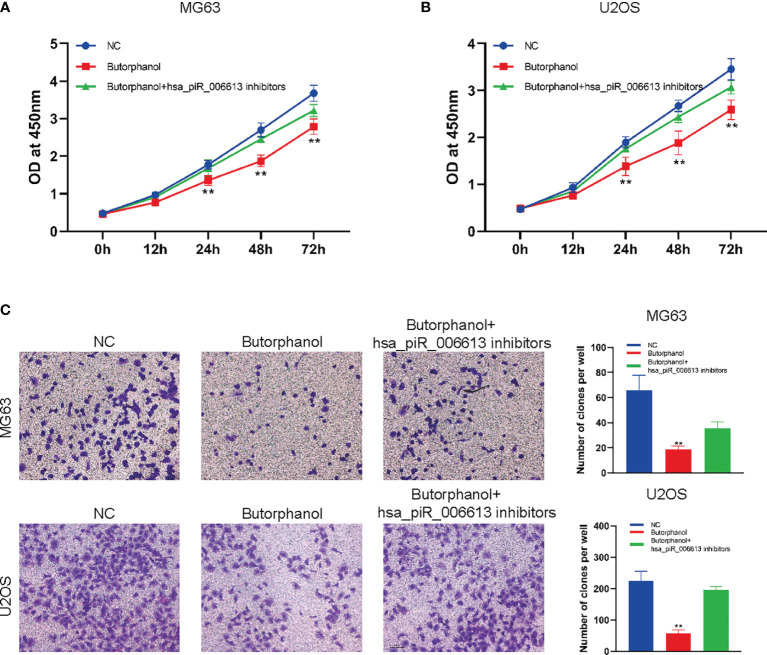
Butorphanol regulates the proliferation and migration of osteosarcoma in a piRNA hsa_piR_006613-dependent manner. **(A)** CCK-8 assay of the viability of MG63 cells treated with butorphanol and hsa_piRNA_006613 inhibitors. **(B)** CCK-8 assay of the viability of U2OS cells treated with butorphanol and hsa_piRNA_006613 inhibitors. **(C)** Transwell assay of the migration of U2OS and MG63 cells treated with butorphanol and hsa_piRNA_006613 inhibitors. ** indicates p<0.01.

To further confirm whether butorphanol regulates osteosarcoma cells through piRNA hsa_piR_006613, hsa_piR_006613 inhibitors were used to inhibit the function of hsa_piR_006613. Results showed that inhibition of has_piR_006613 reduced the effect of butorphanol on the proliferation and migration of osteosarcoma cells (**Figures 5A–C**).

### piRNA hsa_piR_006613 Suppresses the Proliferation of Osteosarcoma *In Vivo*

**Figure 6:**
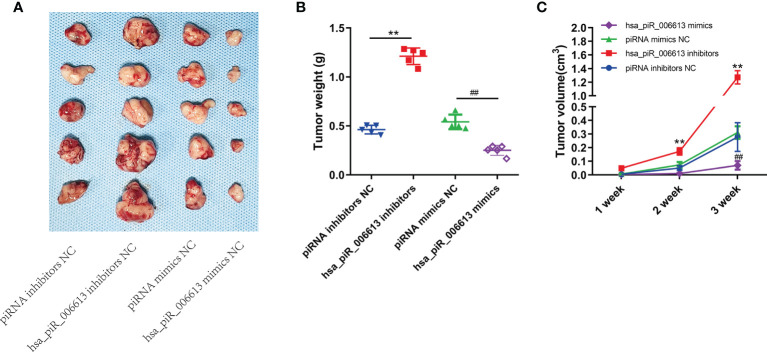
piRNA hsa_piR_006613 suppresses the proliferation of osteosarcoma *in vivo.* **(A)** Subcutaneous injection of U2OS cells with piRNA hsa_piR_006613 overexpression or inhibition into xenograft tumors. **(B)** Tumor weight of xenograft tumors. **(C)** Tumor volume of xenograft tumors. ** indicates compared to piRNA inhibitors NC group p<0.01, ^##^ indicates compared to piRNA mimics NC group p<0.01.

To detect the effect of hsa_piR_006613 on the proliferation of osteosarcoma cells *in vivo*, we constructed a mouse subcutaneous ectopic tumor formation model. Hsa_piR_006613 overexpression reduced the proliferation of osteosarcoma cells and hsa_piR_006613 inhibition promoted the proliferation of osteosarcoma cells *in vivo* (**Figures 6A–C**).

### Butorphanol Downregulated the Expression of FN1 in a piRNA hsa_piR_006613-Dependent Manner

**Figure 7:**
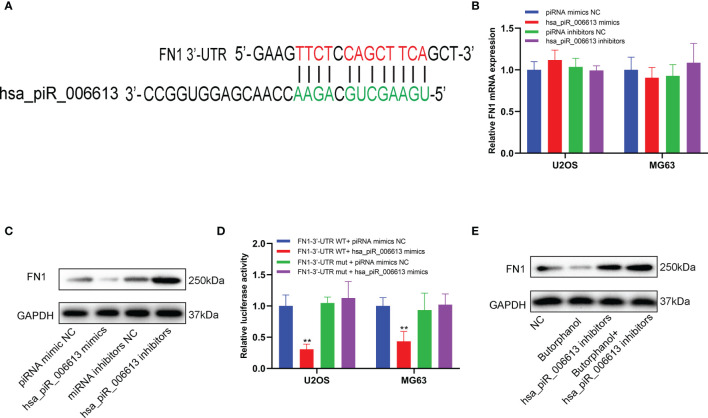
piRNA hsa_piR_006613 suppresses the expression of FN1. **(A)** Software predicted the binding site between piRNA hsa_piR_006613 and FN1 mRNA 3’-UTR. **(B)** The expression of FN1 mRNA in U2OS and MG63 cell transfected with hsa_piR_006613 mimics or inhibitors analyzed by qRT-PCR. **(D)** The expression of FN1 protein in U2OS and MG63 cell transfected with hsa_piR_006613 mimics or inhibitors analyzed by western blot. **(E)** The luciferase assay confirmed that FN1 was the target of piRNA hsa_piR_006613. **(F)** Western blot analyzed the protein expression of FN1 in U2OS cell co-treatment with butorphanol and hsa_piR_006613 inhibitor. Data were expressed as mean ± S.D. of three independent experiments. “**” indicates P<0.01.

The source of piR_hsa_006613 was piRNABank (http://pirnabank.ibab.ac.in/index.shtml) and the sequence was 5’-UGAAGCUGCAGAACCAACGAGGUGGCC-3’. Bioinformatics analysis showed that a putative binding site was found between hsa_piR_006613 and 3’-UTR of FN1 mRNA (**Figure 7A**). The mRNA expression of FN1 was not affected by the altered expression of hsa_piR_006613 (**Figure 7B**). However, the protein expression of FN1 was decreased when hsa_piR_006613 was overexpressed while it was increased when hsa_piR_006613 was knocked down (**Figure 7C**). Luciferase Reporter assay showed that hsa_piR_006613 binded with 3’-UTR of FN1 mRNA in U2OS and MG63 cells (**Figure 7D**). When the expression of hsa_piR_006613 was knockdown, the impact of butorphanol on FN1 protein was reduced (**Figure 7E**). The results indicated that the regulation of butorphanol on FN1 was dependented on piRNA hsa_piR_006613.

## Discussion

Malignant tumors are the main cause of the high incidence rate and mortality worldwide. Even if surgery aims to remove the primary lesion, recurrence and metastasis are the main causes of death. As the main method to treat malignant tumors, surgery may also cause tumor recurrence and metastasis. The possibility of tumor metastasis depends on the balance between the tendency of tumor metastasis and the body’s defense function against cell metastasis. As an important part of surgery and postoperative analgesia, whether anesthesia and analgesia affect tumor recurrence and metastasis has become a research hotspot for anesthetists in recent years. Animal experiments have suggested that the opioids morphine and fentanyl promote tumor metastasis and recurrence. Mathew et al. found that the expression of opioid μ-receptors in non-small cell lung cancer cells is 5–10 times higher than that in normal lung tissue (10). Morphine promotes the growth of Lewis lung cancer cells *in vitro*. Opioid μ-receptor blockers or inhibitors inhibit the proliferation and migration of 50% to 80% of Lewis lung cancer cells (10). *In vitro*, morphine, an opioid commonly used clinically, inhibits the formation of peroxides and expression of cytokines, macrophage function, and NK cell activity. Morphine increases COX-2 expression, promotes the release of prostaglandins, and then promotes tumor growth and metastasis (11). Opioids also have no positive effect on tumor recurrence and metastasis. They inhibit the activity of some immune cells and supress the immune monitoring of tumors (12). However, in the present study, butorphanol effectively inhibited the proliferation and migration of osteosarcoma cells. However, no study has reported the effect of butorphanol on osteosarcoma metastasis *in vivo*. Butorphanol, a commonly used analgesic, must be further studied regarding its immune function in patients with osteosarcoma and its function in osteosarcoma cells.

PiRNA specifically binds to piwi subfamily proteins in the argonaut protein family, which guides the piwi protein and its related epigenetic mechanism to program the genome or transcriptome by identifying many piRNA complementary sequences (7, 13, 14). These activities result in the transcriptional silencing of specific target genes while helping to maintain DNA integrity and tumor stem cell differentiation, epigenetic regulation and embryonic development and disease occurrence and development. In the present study, butorphanol regulates the expression of piRNA in osteosarcoma cells and the regulation of the function of osteosarcoma cells by butorphanol is piRNA dependent. PiRNA-piR651 was reported to be overexpressed in several human cancer tissues compared with paired adjacent normal tissues, including gastric cancer, lung cancer, mesothelial carcinoma, cervical cancer, colon cancer, liver cancer, breast cancer and multiple myeloma tissue. These findings suggest that pir651 may play a carcinogenic role. Recent studies have also found that piR651 improves the clinical diagnosis and treatment of non-small cell lung cancer. PiR36712 inhibits breast cancer metastasis and chemotherapy resistance by regulating the function of SEPW1 (15) and piRNA participate in the metastasis of pancreatic cancer simultaneously (16), and piR54265 promotes the proliferation and migration of colorectal cancer (17). Additionally, a clear cell renal cell carcinoma (ccrcc) study revealed that some characteristics of specific piRNAs can be used as prognostic predictors. Using piRNA microarrays and further identifying candidate piRNAs in a larger cohort, Busch et al. identified three piRNAs significantly associated with tumor recurrence and overall survival: piR-30924, piR-57125 and piR-38756. PiRNA may become a tumor marker through its disease-related biological role and may also be used as a potential clinical therapeutic tool in the future. PiRNA hsa_piR_006613 inhibited the proliferation and migration of osteosarcoma cells and inhibited the proliferation of osteosarcoma cells *in vivo*.

Butorphanol was a commonly used anesthetic and analgesic in clinic, but its regulatory effect on the function of osteosarcoma cells was unknown. In this study, we found that butorphanol could inhibit the proliferation and migration of osteosarcoma cells *in vitro*. In this study, we confirmed that butorphanol could regulate the function of osteosarcoma cells. However, the regulatory mechanism of butorphanol on tumor cell function was unknown. Through sequencing analysis, we found that butorphanol could regulate the expression of piRNA in osteosarcoma cells. At the same time, piRNA hsa_piR_006613 sensitive to butorphanol was found for the first time. We further confirmed the regulatory effect of piRNA on osteosarcoma cells. We also reported for the first time that piRNA hsa_piR_006613 could regulate the proliferation and migration of osteosarcoma cells *in vitro.* However, the mechanism by which hsa_piR_006613 regulates the function of osteosarcoma cells needs to be further studied. It is reported that piRNA can combine with the 3’- UTR region of mRNA to regulate mRNA translation (18). Through bioinformatics research, we found hsa_piR_006613 could bind to the 3’- UTR region of FN1 mRNA. Our results showed that hsa_piR_ 006613 could bind to the 3’- UTR region of FN1 mRNA and inhibit the expression of FN1 protein. Several studies have reported that FN1 promotes the proliferation and metastasis of osteosarcoma cells (19–21). Has_piR_006613 may become a new therapeutic target for osteosarcoma.

## Conclusion

Butorphanol inhibits the proliferation and migration of osteosarcoma cells and can regulate the expression of piRNA hsa_piR_006613 in osteosarcoma cells. hsa_piR_006613 can inhibit the proliferation and migration of osteosarcoma cells. Butorphanol regulates the function of osteosarcoma cells in a piRNA hsa_piR_006613-dependent manner. Hsa_piR_006613 downregulated FN1 protein expression by binding with 3’-UTR of FN1 mRNA. Butorphanol downregulated the expression of FN1 in a piRNA hsa_piR_006613-dependent manner.

## Data Availability Statement

The raw data supporting the conclusions of this article will be made available by the authors, without undue reservation.

## Ethics Statement

The animal study was reviewed and approved by Ethics committee of Yantaishan Hospital.

## Author Contributions

PC, DX, and FL designed and conducted the cell and animal experiments. YG and LD participated in the data analysis, performed the statistical analysis, and drafted the manuscript. All authors read and approved the final manuscript.

## Funding

This project was supported by National Natural Science Foundation of China (NO.81904017).

## Conflict of Interest

The authors declare that the research was conducted in the absence of any commercial or financial relationships that could be construed as a potential conflict of interest.

## Publisher’s Note

All claims expressed in this article are solely those of the authors and do not necessarily represent those of their affiliated organizations, or those of the publisher, the editors and the reviewers. Any product that may be evaluated in this article, or claim that may be made by its manufacturer, is not guaranteed or endorsed by the publisher.

## References

[1] Ji J Lin W Vrudhula A Xi J Yeliseev A Grothusen JR. Molecular Interaction Between Butorphanol and Kappa-Opioid Receptor. Anesthesia Analgesia (2020) 131(3):935–42. doi: 10.1213/ANE.0000000000005017 PMC7668422 32701545

[2] Commiskey S Fan LW Ho IK Rockhold RW. Butorphanol: Effects of a Prototypical Agonist-Antagonist Analgesic on Kappa-Opioid Receptors. J Pharmacol Sci (2005) 98(2):109–16. doi: 10.1254/jphs.CRJ05001X 15942128

[3] Zhu Z Zhang W. Efficacy and Safety of Butorphanol Use in Patient-Controlled Analgesia: A Meta-Analysis. Evidence-Based Complementary Altern Med eCAM (2021) 2021:5530441. doi: 10.1155/2021/5530441 PMC8324365 34335812

[4] Czarnecka AM Synoradzki K Firlej W Bartnik E Sobczuk P Fiedorowicz M. Molecular Biology of Osteosarcoma. Cancers (2020) 12(8):2130. doi: 10.3390/cancers12082130 PMC7463657 32751922

[5] Harrison DJ Geller DS Gill JD Lewis VO Gorlick R. Current and Future Therapeutic Approaches for Osteosarcoma. Expert Rev Anticancer Ther (2018) 18(1):39–50. doi: 10.1080/14737140.2018.1413939 29210294

[6] Liu Y Dou M Song X Dong Y Liu S Liu H. The Emerging Role of the piRNA/Piwi Complex in Cancer. Mol Cancer (2019) 18(1):123. doi: 10.1186/s12943-019-1052-9 31399034 PMC6688334

[7] Xu J Yang X Zhou Q Zhuang J Han S. Biological Significance of piRNA in Liver Cancer: A Review. Biomarkers Biochem Indic Exposure Response Susceptibility to Chemicals (2020) 25(6):436–40. doi: 10.1080/1354750X.2020.1794041 32662667

[8] Chalbatani GM Dana H Memari F Gharagozlou E Ashjaei S Kheirandish P. Biological Function and Molecular Mechanism of piRNA in Cancer. Pract Lab Med (2019) 13:e00113. doi: 10.1016/j.plabm.2018.e00113 30705933 PMC6349561

[9] Romano G Veneziano D Acunzo M Croce CM. Small Non-Coding RNA and Cancer. Carcinogenesis (2017) 38(5):485–91. doi: 10.1093/carcin/bgx026 PMC6248440 28449079

[10] Mathew B Lennon FE Siegler J Mirzapoiazova T Mambetsariev N Sammani S. The Novel Role of the Mu Opioid Receptor in Lung Cancer Progression: A Laboratory Investigation. Anesthesia Analgesia (2011) 112(3):558–67. doi: 10.1213/ANE.0b013e31820568af PMC4327979 21156980

[11] Farooqui M Li Y Rogers T Poonawala T Griffin RJ Song CW. COX-2 Inhibitor Celecoxib Prevents Chronic Morphine-Induced Promotion of Angiogenesis, Tumour Growth, Metastasis and Mortality, Without Compromising Analgesia. Br J Cancer (2007) 97(11):1523–31. doi: 10.1038/sj.bjc.6604057 PMC2360252 17971769

[12] Cheng WF Chen LK Chen CA Chang MC Hsiao PN Su YN. Chimeric DNA Vaccine Reverses Morphine-Induced Immunosuppression and Tumorigenesis. Mol Ther J Am Soc Gene Ther (2006) 13(1):203–10. doi: 10.1016/j.ymthe.2005.06.479 16140583

[13] Czech B Munafo M Ciabrelli F Eastwood EL Fabry MH Kneuss E. piRNA-Guided Genome Defense: From Biogenesis to Silencing. Annu Rev Genet (2018) 52:131–57. doi: 10.1146/annurev-genet-120417-031441 PMC10784713 30476449

[14] Lenart P Novak J Bienertova-Vasku J. PIWI-piRNA Pathway: Setting the Pace of Aging by Reducing DNA Damage. Mech Ageing Dev (2018) 173:29–38. doi: 10.1016/j.mad.2018.03.009 29580825

[15] Tan L Mai D Zhang B Jiang X Zhang J Bai R. PIWI-Interacting RNA-36712 Restrains Breast Cancer Progression and Chemoresistance by Interaction With SEPW1 Pseudogene SEPW1P RNA. Mol Cancer (2019) 18(1):9. doi: 10.1186/s12943-019-0940-3 30636640 PMC6330501

[16] Li F Yuan P Rao M Jin CH Tang W Rong YF. piRNA-Independent Function of PIWIL1 as a Co-Activator for Anaphase Promoting Complex/Cyclosome to Drive Pancreatic Cancer Metastasis. Nat Cell Biol (2020) 22(4):425–38. doi: 10.1038/s41556-020-0486-z 32203416

[17] Mai D Ding P Tan L Zhang J Pan Z Bai R. PIWI-Interacting RNA-54265 Is Oncogenic and a Potential Therapeutic Target in Colorectal Adenocarcinoma. Theranostics (2018) 8(19):5213–30. doi: 10.7150/thno.28001 PMC6276099 30555542

[18] Han H Fan G Song S Jiang Y Qian C Zhang W. piRNA-30473 Contributes to Tumorigenesis and Poor Prognosis by Regulating M6a RNA Methylation in DLBCL. Blood (2021) 137(12):1603–14. doi: 10.1182/blood.2019003764 32967010

[19] Zhou Y Yin L Li H Liu LH Xiao T. The LncRNA LINC00963 Facilitates Osteosarcoma Proliferation and Invasion by Suppressing miR-204-3p/FN1 Axis. Cancer Biol Ther (2019) 20(8):1141–8. doi: 10.1080/15384047.2019.1598766 PMC6605988 30975024

[20] Saba KH Cornmark L Rissler M Fioretos T Astrom K Haglund F. Genetic Profiling of a Chondroblastoma-Like Osteosarcoma/Malignant Phosphaturic Mesenchymal Tumor of Bone Reveals a Homozygous Deletion of CDKN2A, Intragenic Deletion of DMD, and a Targetable FN1-FGFR1 Gene Fusion. Genes Chromosomes Cancer (2019) 58(10):731–6. doi: 10.1002/gcc.22764 31066955

[21] Kun-Peng Z Chun-Lin Z Xiao-Long M Lei Z. Fibronectin-1 Modulated by the Long Noncoding RNA OIP5-AS1/miR-200b-3p Axis Contributes to Doxorubicin Resistance of Osteosarcoma Cells. J Cell Physiol (2019) 234(5):6927–39. doi: 10.1002/jcp.27435 30204936

